# Functional analysis of a viral promoter from a strawberry vein banding virus isolate from China

**DOI:** 10.1186/s12985-022-01778-2

**Published:** 2022-03-31

**Authors:** Lei Jiang, Jing Chen, You-zhi Yang, Rui Li, Shuang Li, Zhan-qi Wang, Tong Jiang

**Affiliations:** 1 School of Plant Protection, Anhui Agricultural University, Hefei, 230036 People’s Republic of China; 2 Key Laboratory of Vector Biology and Pathogen Control of Zhejiang Province, College of Life Sciences, Huzhou University, Huzhou, 313000 People’s Republic of China; 3 Anhui Province Key Laboratory of Integrated Pest Management on Crops, Hefei, 230036 People’s Republic of China; 4 Key Laboratory of Biology and Sustainable Management of Plant Diseases and Pests of Anhui Higher Education Institutes, Anhui Agricultural University, Hefei, 230036 People’s Republic of China

**Keywords:** Strawberry vein banding virus, Chinese isolate, Full-length promoter, Promoter activity, Cauliflower mosaic virus 35S promoter

## Abstract

**Background:**

Promoters are important factors affecting gene expression in cells. The driven activities of viral promoters were generally assessed to screen available promoters for transgenic and research and biotech industries. In this study, we cloned a full-length promoter from a Chinese isolate of strawberry vein banding virus (SVBV) and produced several deletion mutants for evaluation of applications in production of reporter proteins in stable transgenic plants.

**Methods:**

The full-length promoter of SVBV (SP1) and its three deletion mutants (SP2, SP3, and SP4) were amplified using polymerase chain reaction. The effects of SVBV SP1, SP2, SP3, and SP4 on gene expression were evaluated using β-glucuronidase (*GUS*) and green fluorescent protein (*GFP*) reporter genes.

**Results:**

Transient expression assays showed that the SVBV SP1 promoter and its three deletion mutants all expressed the reporter genes, albeit at very different levels. Interestingly, transcriptional activity driven by the SP1 promoter was much higher than that of the cauliflower mosaic virus (CaMV) 35S promoter. After stable transformation of the *GUS* gene into *Nicotiana tabacum* plants, SVBV SP1-driven transgene expression was approximately 2.6-fold higher than CaMV 35S promoter-driven transgene expression. In addition, *GUS* gene expression levels were enhanced by co-inoculation of the plants with the SP1 promoter-driven vector carrying the *GUS* gene and the vector expressing SVBV open reading frame (ORF) V or ORF VI.

**Conclusions:**

The SVBV SP1 promoter from the Chinese isolate evaluated in this study could successfully drive transient and stable expression in plants, it was a stronger promoter than the CaMV 35S and FLt-US promoters and may be more useful for the production of stable transgenic plants.

**Supplementary Information:**

The online version contains supplementary material available at 10.1186/s12985-022-01778-2.

## Background

The cauliflower mosaic virus (CaMV) 35S promoter is a strong foreign gene expression promoter that has been successfully used to study gene functions in many dicotyledonous and monocotyledonous species through *Agrobacterium*- or particle bombardment-mediated transformation technologies. Using this plant virus-derived promoter, numerous transgenic crop plants, including cotton, soybean, rice, and many other species, with improved resistance against insects, diseases, and abiotic stresses have been produced [1–6].

The CaMV 35S promoter is currently the most widely used gene expression promoter for stable plant transformation, including that in strawberries. Although earlier studies showed that the CaMV 35S promoter can facilitate strong expression of foreign genes in plants, recent studies have indicated that its expression activity is lower than those of several plant-derived expression promoters [7–10]. For example, strawberry plants transformed with an RNA silencing vector expressing an antisense fragment of a pectate lyase gene through a 35S promoter showed only a 30% reduction in endogenous pectate lyase gene expression in strawberry plants, leading to no clear phenotype change in fruit color, size, shape, and weight [7]. Owens et al. reported the expression of a cold-inducible transcription factor, *CBF1*, in strawberry plants [10]. Reverse transcription quantitative polymerase chain reaction (RT-qPCR) analysis of two stable transgenic strawberry lines showed that the expression level of the transgene driven by the 35S promoter was very weak in both leaves and receptacles. Consequently, the resulting transgenic plants did not show a clear improvement in cold tolerance [10]. In 2001, Zhang et al. published a transformation protocol for strawberries; the authors transformed the strawberry variety ‘Tudla’ with the β-glucuronidase (*GUS*) gene driven by the 35S promoter, and only 50% of transgenic plants showed GUS activity [9].

The level of foreign gene expression in transgenic plants is mainly dependent on the strength of the promoter in the expression vector. Promoters are recognized by RNA polymerases, followed by the initiation of gene transcription. The 35S promoter was originally identified in the genome of CaMV, a virus known to primarily infect *Brassicaceae* plants. Although both strawberry vein banding virus (SVBV) and CaMV belong to the genus *Caulimovirus*, family *Caulimoviridae* [11–13], SVBV mainly infects strawberries and causes yellow vein banding and leaf twisting symptoms [11, 12, 14]. The genome of SVBV is a circular double-stranded DNA of approximately 8 kb in length, containing seven open reading frames (ORFs) [12]. The arrangement of the SVBV genome structure resembles that of CaMV, and the sequence of the SVBV promoter, which is located downstream of ORF VI, is similar to that of the CaMV 35S promoter. In 2000, Wang et al. cloned the full-length SVBV promoter and used it to express a full-length infectious clone of zucchini yellow mosaic virus in cucumber, melon, squash, and watermelon [15]. Moreover, in 2004, Pattanaik et al. further characterized the promoter from an SVBV isolate from the United States of America (USA) and found that a 371-bp fragment from the 3′ half (− 352 to + 19 from the transcription start site) of the promoter conferred maximum transcriptional activity during protoplast assays [16].

In this study, we cloned the full-length promoter from an SVBV isolate from China (hereafter referred to as SVBV SP1), compared its expression activity with the promoter of the SVBV USA isolate, and analyzed the key elements inside the promoter to assess their effects on enhancement of gene expression. We propose the use of this promoter for foreign gene expression in transgenic strawberries or other *Rosaceae* plants. Our results also provided evidence showing that both SVBV ORF V and VI products enhanced foreign gene expression driven by the SVBV SP1 promoter in plants.

## Methods

### Construction of expression vectors

DNA from the Chinese SVBV isolate was obtained from an SVBV-infected strawberry leaf sample. The promoter of this virus isolate and three deletion mutants were generated by PCR amplification using viral DNA-specific primers (Table 1). The PCR-amplified fragments were individually cloned into a pGEM-T vector (Promega, Madison, WI, USA). After DNA sequencing, the correct fragments were released from the pGEM-T cloning vectors through double digestion with *Bam*HI, *Xba*I, *Eco*RI, and *Bam*HI restriction enzymes. The double digested fragments were then inserted individually into the *Bam*HI*/Xba*I or *Eco*RI/*Bam*HI sites within the binary vector pINT121 or pCHF3 to generate pINT-SP1, pINT-SP2, pINT-SP3, pINT-SP4, pCHF-SP1, pCHF-SP2, pCHF-SP3, and pCHF-SP4 constructs. The pINT constructs contained a *GUS* gene, and the pCHF constructs contained a green fluorescent protein (*GFP*) gene. For comparison, the full-length SVBV-E3 promoter was PCR-amplified from the plasmid pSVBV-E3 (a gift from Prof. Stenger DC, University of Nebraska, Lincoln, NE, USA) using the primer pair FLtUSCH-F-1/FLtUSCH-R-1 or FLtUSCH-F-2/FLtUSCH-R-2 (Table 1). The resulting fragments were also inserted into the vector pINT121 or pCHF3 to produce pINT-FLt-US or pCHF-FLt-US. The expression vectors were transformed individually into *Agrobacterium tumefaciens* strain EHA105 cells, as described previously [17], and used for transient or stable expression assays.

**Table 1:** Primer pairs used in this study

Name of primers	Sequence of primers	Restriction site
P1/F	5′-**GGATCC**GTCATCGCATATGTTCGAGACC-3′	*Bam*HI
P1/R	5′-**TCTAGA**ATGTAAGCAGTTAGGCCCTGTG-3′	*Xba*I
P2/F	5′-**GGATCC**CATGGACTCCTTGACTATGTACA-3′	*Bam*HI
P2/R	5′-**TCTAGA**CCGGCAGTTCTTGACTAGGACCT-3′	*Xba*I
P3/F	5′-**GGATCC**GTCATCCAAAGAGCACTTAGACC-3′	*Bam*HI
P3/R	5′-**TCTAGA**CGTAGCTACGTACCCCGATGGC-3′	*Xba*I
P4/F	5′-**GGATCC** GTCATCGCATATGTTCGAGACC-3′	*Eco*RI
P4/R	5′-**TCTAGA**ATGTAAGCAGTTAGGCCCTGTG-3′	*Bam*HI
USPCH/F-1	5′-**GGATCC**AGAGCACTTCCAAAGA-3′	*Bam*HI
USPCH/R-1	5′-**TCTAGA**GTTAGGTAAGCAGCTA-3′	*Xba*I
CHPCH/F1	5′-**GAATTC**GTCATCCAAAGAGCACTTAGACC-3′	*Eco*RI
CHPCH/R1	5′-**GGATCC**CGTAGCTACGTACCCCGATGGC-3′	*Bam*HI
CHPCH/F2	5′-**GAATTC**CATGGACTCCTTGACTATGTACA-3′	*Eco*RI
CHPCH/R2	5′-**GGATCC**CCGGCAGTTCTTGACTAGGACCT-3′	*Bam*HI
CHPCH/F3	5′-**GAATTC**GTCATCCAAAGAGCACTTAGACC-3′	*Eco*RI
CHPCH/R3	5′-**GGATCC** CGTAGCTACGTACCCCGATGGC-3′	*Bam*HI
CHPCH/F4	5′-**GAATTC**GTCATCGCATATGTTCGAGACC-3′	*Eco*RI
CHPCH/R4	5′-**GGATCC**ATGTAAGCAGTTAGGCCCTGTG-3′	*Bam*HI
USPCH/F-2	5′-**GAATTC**AGAGCACTTCCAAAGA-3′	*Eco*RI
USPCH/R-2	5′-**GGATCC**GTTAGGTAAGCAGCTA-3′	*Bam*HI
GUS/F	5′-CATGGCTGGATATGTATCACCGCGT-3′	–
GUS/R	5′-CGAAGTTCATGCCAGTCCAGCGT-3′	–
β-actin/F	5′-CAATCCAGACACTGTACTTTCTCTC-3′	–
β-actin/R	5′-AAGCTGCAGGTATCCATGAGACTA-3′	–

### Transient gene expression assays and stable plant transformation

Transient gene expression assays were conducted through *Agrobacterium*-mediated inoculation, as previously described [18]. Briefly, *Agrobacterium* cells containing one of the expression vectors were grown until reaching an optical density at 600 nm (OD_600_) of 0.8. The cells were then pelleted and resuspended in an inoculation solution (10 mM 2-morpholinoethane-sulfonic acid, 10 mM MgCl_2_, and 100 µM acetosyringone) to an OD_600_ of 1.5. Next, the *Agrobacterium* suspension (500 µL) harboring a specific expression vector was inoculated into leaves or injected into the stems of an *N. benthamiana* plant using one ml syringes. The inoculated leaves were harvested at 64 h postinoculation (hpi) and analyzed individually for GUS activity using a fluorometric assay or for GFP signals under a confocal microscope. For *Agrobacterium*-injected stems, stem cross-sections were cut just above the injection site and used for histochemical staining assays. Three independent experiments were conducted for each construct. *Agrobacterium*-mediated stable transformation of *Nicotiana tabacum* was performed using the leaf disc transformation method, as previously reported [19]. Positive transgenic plants were selected on MS medium containing 100 µg/mL kanamycin and 500 µg/mL carbenicillin, followed by PCR confirmation of the selected plants using *GUS* or *GFP* gene-specific primers.

### Analyses of *GUS* gene expression using histochemical and fluorometric assays

Histochemical assays were performed as previously described [20], with minor modifications. Briefly, free-hand cut sections were prepared from plant roots, stems, or leaves and then incubated overnight in a GUS staining solution (1 mM 5-bromo-4-chloro-3-indolyl-β-d-glucuronic acid in 50 mM sodium phosphate buffer, pH 7.0) at 37 °C. Sections (4–6 µm thick) were cut from the embedded tissues using a 11,800 LKB Pyramitome (LKB-BROMMA, Stockholm, Sweden). Images of the sections were captured using a Leica DC300 stereomicroscope (Leica, Mannheim, Germany). For fluorometric assays, *N. benthamiana* leaves were sampled and homogenized in passive lysis buffer (Promega). After centrifugation, supernatants were collected and used for fluorometric assays, as described previously [21]. Fluorescence from 4-methylumbelliferone (MU), a cleavage product of 4-methylumbelliferyl-β-d-glucuronide (MUG), was measured using a luminescence spectrometer (LS50B; Perkin-Elmer) with excitation and emission wavelengths of 365 and 455 nm, respectively. The protein contents in these samples were estimated using an Eppendorf BioPhotometer (Eppendorf, Hamburg, Germany). GUS activity in each sample was calculated as the amount of MU released from MUG in pmol/min/µg protein. The mean GUS activity in pINT121 vector-transformed tissues was set at 100% and was used to normalize the GUS activities from other tissue samples. The significance of differences between samples was analyzed using the LSD method in SPSS 12.0.

### RT-qPCR analysis

Total RNA was isolated from transgenic tobacco seedlings harboring the pINT-SP1, pINT-FLt-US, or pINT121 expression vector using TRIzol reagent (Invitrogen, CA, USA), followed by DNase I treatment. First-strand cDNA was synthesized using 1 mg total RNA per 20-µL RT reaction and an AMV RNA PCR Kit (TaKaRa, Dalian, China). qPCR was performed using a SYBR Premix Ex Tap II kit (TaKaRa), according to the manufacturer’s instructions. The expression level of the *GUS* gene in the transgenic tobacco lines was determined by RT-qPCR using gene-specific primers (Table 1). The expression level of the tobacco *β-actin* gene was used as an internal control during the assay, as described previously [18].

### Co-inoculation of *N. benthamiana* leaves with the SVBV SP1 promoter and one of the SVBV protein expression constructs

Individual ORFs of the Chinese SVBV isolate were amplified by PCR and cloned individually behind a CaMV 35S promoter inside the pBIN-PLUS vector to generate pBIN-ORFI, pBIN-ORFII, pBIN-ORFIII, pBIN-ORFIV, pBIN-ORFV, and pBIN-ORFVI vectors. The resulting constructs were transformed individually into *A. tumefaciens* cells and co-inoculated into *N. benthamiana* leaves with an *Agrobacterium* harboring the pINT-SP1 vector. The activities of the *GUS* gene in these inoculated leaves were determined at 64 hpi through fluorometric assays, as described by Zhang et al. [21].

## Results

### Construction of expression vectors harboring the full-length or partial SVBV promoters

**Fig. 1:**
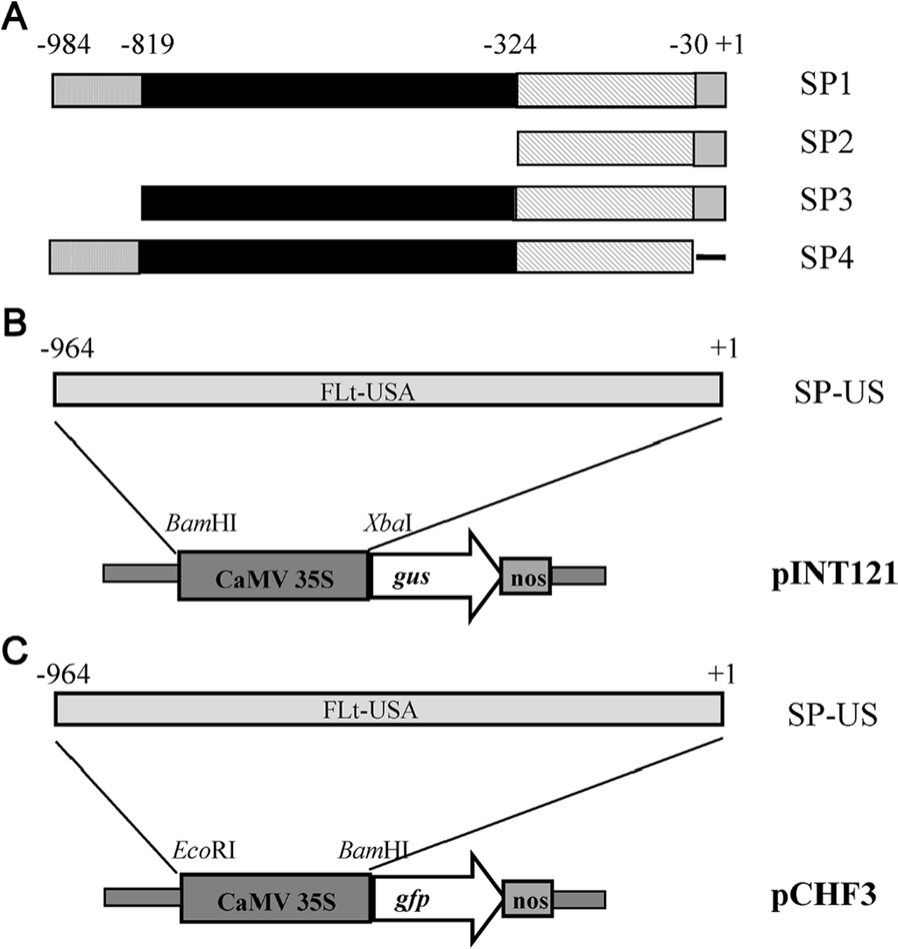
Schematic representations of the expression vectors. **A** The full-length SVBV SP1 promoter and its deletion mutants. **B** Schematic illustration of pINT-FLt-US. **C** Schematic illustration of pCHF-FLt-US. SP1 is a full-length SVBV Chinese isolate promoter and mutant SP2 promoter contained a fragment of SP1, ranging from nucleotides − 324 bp ~  + 1, and retained only the downstream core promoter region with a GA-motif and two CAAT-boxes. Mutant SP3 promoter had a deletion from nucleotide position − 984 ~ -819 bp and thus represented the upstream regulatory elements plus the core promoter region. Mutant SP4 promoter contained the full-length promoter sequence except the 30 nucleotides upstream of the transcription initiation site (+ 1)

The full-length SVBV promoter from the Chinese SVBV isolate (GenBank accession number HE681085) was amplified by PCR. Sequence analysis using the Plant CARE program [22] showed that the full-length SVBV promoter contained several typical cis-acting elements, such as a TATA box, CAAT box, and potential cis-regulatory elements, including GATA motifs and TC-rich repeats. In previous research, important domains of viral promoters were generally incorporated to generate different mutants, and the driven activities were assessed in tobacco and other plants [23]. According to this prediction, a full-length SVBV promoter from the Chinese isolate (SP1) and three deletion mutants (SP2, SP3, and SP4) were constructed (Fig. 1A). The position of the SP1 promoter in the SVBV genome is shown. The mutant SP2 promoter contained a fragment of SP1, ranging from nucleotides − 324 to + 1 bp, and this mutant retained only the downstream core promoter region, which harbored a GA motif and two CAAT boxes. The mutant SP3 promoter had a deletion from nucleotide positions − 984 to − 819 bp and thus represented the upstream regulatory elements plus the core promoter region. The mutant SP4 promoter contained the full-length promoter sequence, except the 30 nucleotides upstream of the transcription initiation site (+ 1). Full-length and mutant SVBV promoters were individually used to replace the 35S promoter in the pCHF3 or pINT121 expression vector to produce pCHF-SP1, pCHF-SP2, pCHF-SP3, pCHF-SP4, pINT-SP1, pINT-SP2, pINT-SP3, and pINT-SP4. The expression vectors pCHF3 and pINT121 without their original 35S promoter (pCHF3-35SΔ and pINT121-35SΔ) served as negative control vectors. In this study, the full-length promoter of the SVBV isolate from the USA (SP-US) was also amplified by PCR and used to replace the 35S promoter in pCHF3 and pINT121 to generate pCHF-FLt-US and pINT-FLt-US, respectively. These two vectors were also used for comparisons in this study (Fig. 1B, C).

### The full-length SVBV SP1 promoter showed potential for transient expression of exogenous genes in plants

**Fig. 2:**
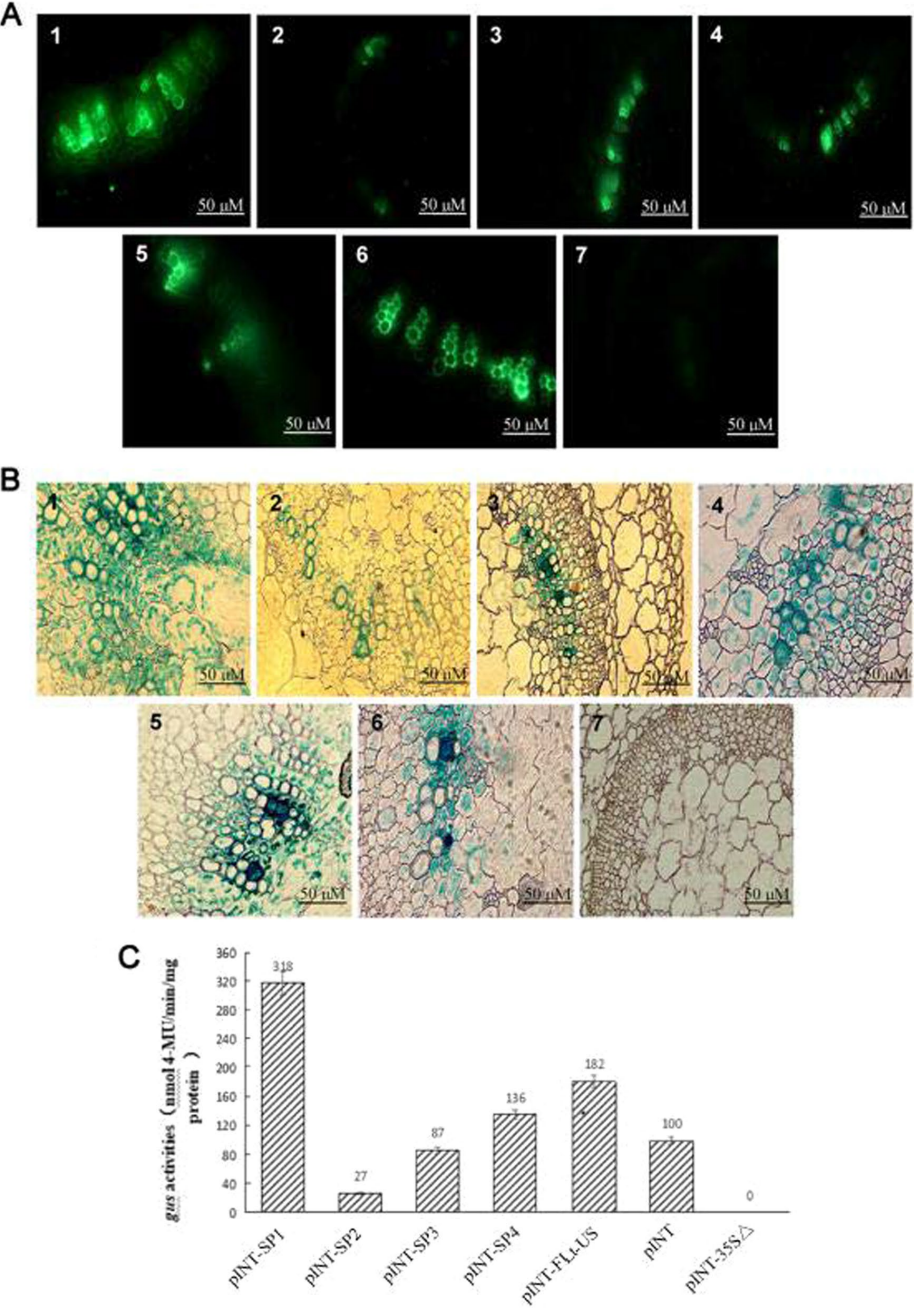
The full-length SVBV SP1 promoter showed potential for transient expression of exogenous genes in plants. **A** Transient expression of *GFP* in *N. benthamiana* stems. *Agrobacterium* cells harboring the pCHF-SP1 (1), pCHF-SP2 (2), pCHF-SP3 (3), pCHF-SP4 (4), pCHF-FLt-US (5), pCHF3 (6), or pCHF3-35SΔ (7) vector were injected into the stems of *N. benthamiana* plants. Freehand sections were cut from the injected stems at 64 hpi, and GFP signals were detected under a fluorescent microscope. Bar = 50 µM. **B** Analysis of *GUS* gene expression in *N. benthamiana* stems. *Agrobacterium* cells harboring the pINT-SP1 (1), pINT-SP2 (2), pINT-SP3 (3), pINT-SP4 (4), pINT-FLt-US (5), pINT121 (6), or pINT-35SΔ (7) vector were injected into the stems of *N. benthamiana* plants. The injected stems were harvested at 64 hpi and stained overnight in a 1 mM X-Gluc staining solution prior to paraffin embedding. Sections prepared from the embedded tissues were examined under a microscope for GUS staining results. Blue staining indicates positive expression of the *GUS* gene in stems. Bar = 50 µM. **C** Analysis of GUS activity in *N. benthamiana* leaves. *Agrobacterium* cells harboring the pINT-SP1, pINT-SP2, pINT-SP3, pINT-SP4, pINT-FLt-US, pINT121, or pINT-35SΔ (negative control) vector were inoculated into leaves of *N. benthamiana* plants. The inoculated leaves were harvested at 64 hpi and used for GUS activity fluorometric assays. Each treatment had five biological replicates, and the experiment was repeated three times. Standard errors were determined using the LSD method. The biochemical expression assay in leaves was repeated thrice and average readings are represented. Statistical analysis showed a *P* value of < 0.05, indicating high significance

To detect the SP1 promoter activity in transient expression, *Agrobacterium* harboring expression vector pCHF-SP1, pCHF-SP2, pCHF-SP3, pCHF-SP4, pCHF-FLt-US or pCHF3-35SΔ was injected into *N. benthamiana* stems. The injected stems were harvested at 64 hpi, and freehand-cut cross-sections from these stems were examined under a Leica DC300 stereomicroscope. The results of the study showed that GFP-derived green fluorescence appeared exclusively in the vascular tissues of stem sections injected with the expression vectors pCHF-SP1, pCHF-SP2, pCHF-SP3, pCHF-SP4, and pCHF-FLt-US. The fluorescence intensity of GFP driven by the SP1 promoter was significantly stronger than that of the other mutants and was also stronger than that of GFP driven by the CaMV 35S promoter. No green fluorescence was observed in sections from the plants injected with *Agrobacterium* harboring pCHF3-35SΔ (Fig. 2A).

*GUS* genes were then transiently expressed in the stem sections inoculated by *Agrobacterium* harboring the expression vectors pINT-SP1, pINT-SP2, pINT-SP3, pINT-SP4, pINT-FLt-US, and pINT-35SΔ, following injection into the stems of *N. benthamiana* plants. Histochemical staining of sections of paraffin-embedded stem tissues followed by light microscopy showed that the blue staining signal, representing *GUS* gene expression, was mainly localized in the vascular tissues and in some cells in the cortex of all pINT-SP1-, pINT-SP2-, pINT-SP3-, pINT-SP4-, or pINT-FLt-US-injected stem sections. GUS intensity in cells harboring the SP1 promoter was significantly stronger than that of other mutants and stronger than that of 35S-driven GUS. No blue staining was observed in sections from stems injected with *Agrobacterium* harboring the pINT-35SΔ vector (Fig. 2B).

GUS activity was then analyzed in tissues using fluorometric assays. *Agrobacterium* harboring vectors pINT-SP1, pINT-SP2, pINT-SP3, pINT-SP4, pINT-FLt-US, and pINT-35SΔ were inoculated into *N. benthamiana* leaves, and the inoculated leaves were harvested at 3 days postinoculation and analyzed for GUS activity using fluorometric assays. The results showed that the average GUS activity in leaves inoculated with *Agrobacterium* harboring the pINT-SP1 vector was approximately 3.2- and 1.8-fold greater than those in leaves inoculated with *Agrobacterium* harboring the pINT121 and pINT-FLt-US vectors, respectively (Fig. 2C). The mean GUS activity in leaves inoculated with *Agrobacterium* harboring the mutant pINT-SP4 vector was approximately 1.4-fold greater than that in leaves inoculated with *Agrobacterium* harboring the pINT121 vector, but was only approximately 75% that in leaves inoculated with *Agrobacterium* harboring the pINT-FLt-US vector. The mean GUS activity in leaves inoculated with *Agrobacterium* harboring the mutant pINT-SP2 or pINT-SP3 vector was lower than that in leaves inoculated with *Agrobacterium* harboring the pINT121 vector.

### The full-length SVBV SP1 promoter showed potential for stable expression of exogenous genes in plants

**Fig. 3:**
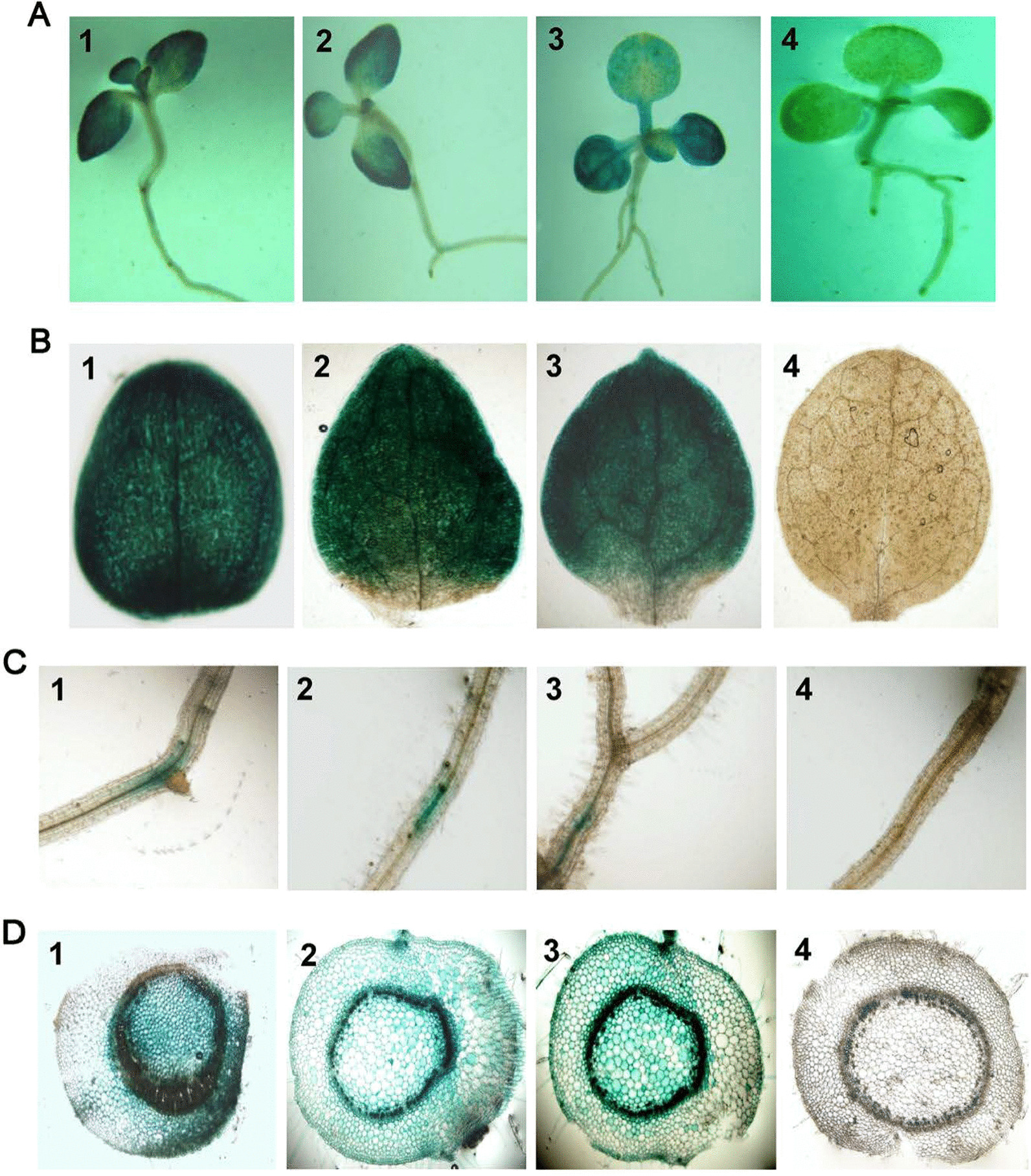
Histochemical assays of *GUS* gene expression in transgenic tobacco seedlings, leaves, roots, and stems. *N. benthamiana* plants transformed with the pINT SP1 (1), pINT-FLt-US (2), pINT121 (3) or pINT-35SΔ (4, negative control) vector were used in these assays. Expression of the *GUS* gene was determined by overnight staining of tissues using 1 mM X-Gluc staining solution. **A** Images of seedlings representing the four different transgenic plant lines are shown. **B** Images of fully expanded leaves harvested from different transgenic plants and stained for GUS activity. **C** Images of roots harvested from different transgenic plants and stained for GUS activity. **D** Images of stem cross sections prepared from different transgenic plants and stained for GUS activity

The strength of promoter activity determines the expression levels of transgenes in plants. To compare the strength of promoter activity among the SVBV SP1, SVBV USA (FLt-US), and CaMV 35S (35S) promoters, tobacco plants were stably transformed with pINT-SP1, pINT-FLt-US, pINT121, or pINT-35SΔ vector, and transgenic tobacco seedlings or leaves were harvested and analyzed for promoter expression using histochemical staining. The results showed that both SVBV SP1 and FLt-US promoters conferred stronger *GUS* gene expression in transgenic tobacco seedlings and expand throughout the whole leaves compared with that in pINT121-transformed tobacco (Fig. 3A, B). Histochemical staining also showed that *GUS* gene expression was mainly observed in vascular bundles and that all transgenic tobacco plants showed *GUS* gene expression in the elongation zone of the roots (Fig. 3C). Furthermore, in stem cross-sections from transgenic plants, *GUS* gene expression was mainly present in the epidermal layer, pith, cortex, and vascular cells (Fig. 3D). These results suggested that the expression strength of the SVBV SP1 promoter was greater than that of the SVBV FLt-US and CaMV 35S promoters.

### Analysis of GUS activity and accumulation of GUS mRNA in transgenic plants

**Fig. 4:**
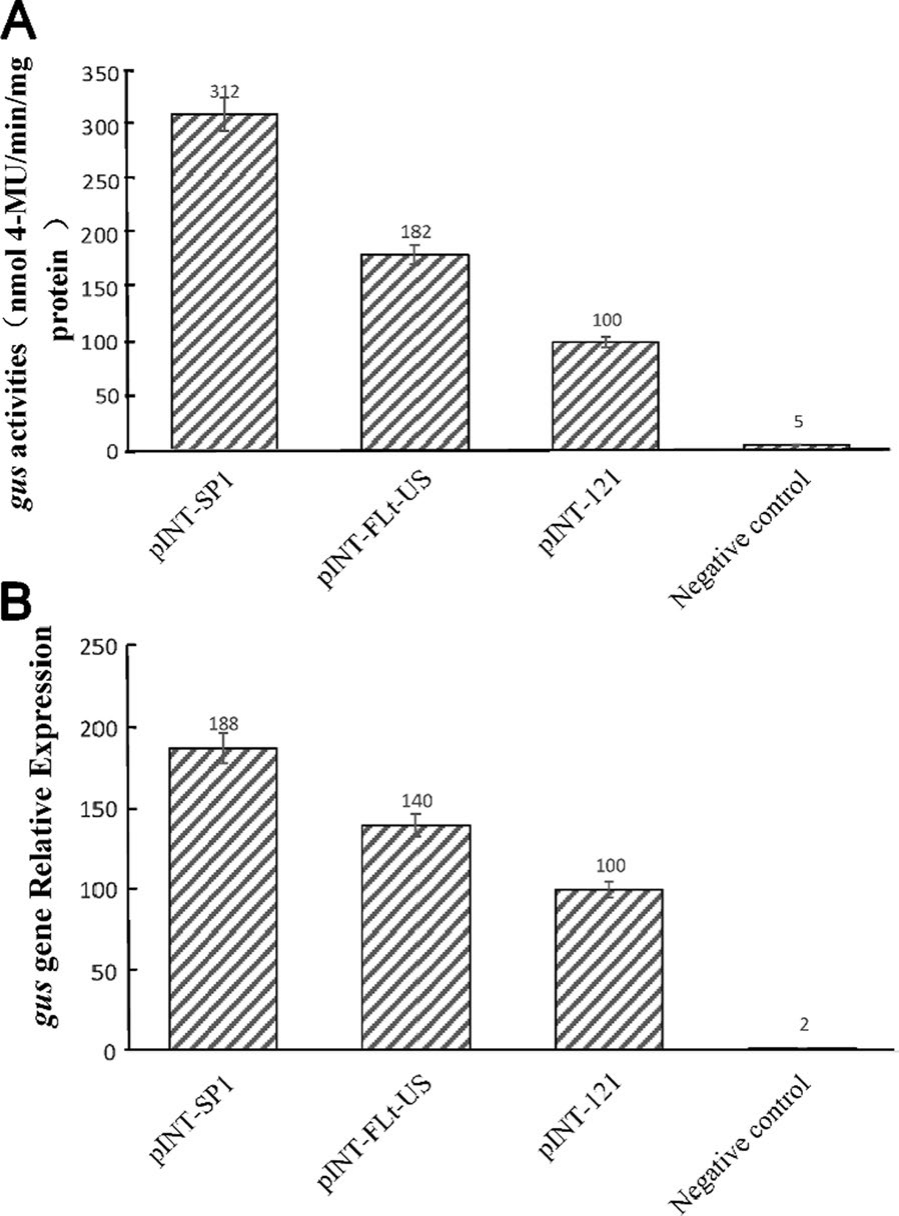
Analysis of GUS activity and accumulation of *GUS* mRNA in transgenic plants. **A** GUS activities in the leaves from different transgenic tobacco plants. Leaves were harvested from tobacco plants transformed with pINT-SP1, pINT-FLt-US, pINT121, or pINT-35SΔ (negative control) vector. The harvested leaf tissues were analyzed for GUS activity using fluorometric assays. Five biological replicates were used for each treatment, and the experiment was repeated three times. Standard errors were determined using the LSD method. The biochemical expression assay in leaves was repeated thrice and average readings are represented. Statistical analysis showed a *P* value of < 0.05, indicating high significance. **B** Relative expression levels of the *GUS* gene in different transgenic tobacco plants. Total RNA was extracted from leaves harvested from tobacco plants transformed with the pINT-SP1, pINT-FLt-US, pINT121, or pINT-35SΔ (negative control) vector. Relative *GUS* mRNA levels were determined by RT-qPCR using *GUS* gene-specific primers. The relative expression level of the tobacco β-actin gene was used as an internal control. Standard errors were determined using the LSD method. The biochemical expression assay in leaves was repeated thrice and average readings are represented. Statistical analysis showed a *P* value of < 0.05, indicating high significance

To confirm that the activity of the SVBV SP1 promoter was stronger than that of the SVBV FLt-US promoter or CaMV 35S promoter, leaves were harvested from plants transformed with the pINT-SP1, pINT-FLt-US, or pINT121 vector and analyzed for GUS activity using fluorometric assays. The results showed that the average GUS activity in leaves harvested from plants transformed with the pINT-SP1 vector was approximately 1.7- and 3.1-fold greater than that in leaves harvested from plants transformed with the pINT-FLt-US or pINT121 vector (Fig. 4A). This finding was consistent with our GUS staining results (Fig. 3) and suggested that the promoter of the Chinese SVBV isolate may be used to generate stable transgenic plants with significantly higher levels of transgene expression than that in the plants transformed with a vector harboring the SVBV FLt-US or CaMV 35S promoter.

To confirm the GUS activity observed in Fig. 4A, we next assessed GUS mRNA levels in different transgenic plants using RT-qPCR of total RNA isolated from tobacco seedlings transformed with the pINT-SP1, pINT-FLt-US, pINT121, and pINT-35SΔ vectors. The results showed that GUS mRNA accumulated to a significantly higher level in pINT-SP1-transformed tobacco seedlings than in pFLt-US- or pINT121-transformed seedlings (Fig. 4B). This result was consistent with the results of GUS activity assays (Fig. 4A) and indicated that the transcriptional activity of the SVBV SP1 promoter was indeed stronger than that of the SVBV FLt-US or CaMV 35S promoter.

### Influence of other SVBV-encoded proteins on SP1 promoter activity

**Fig. 5:**
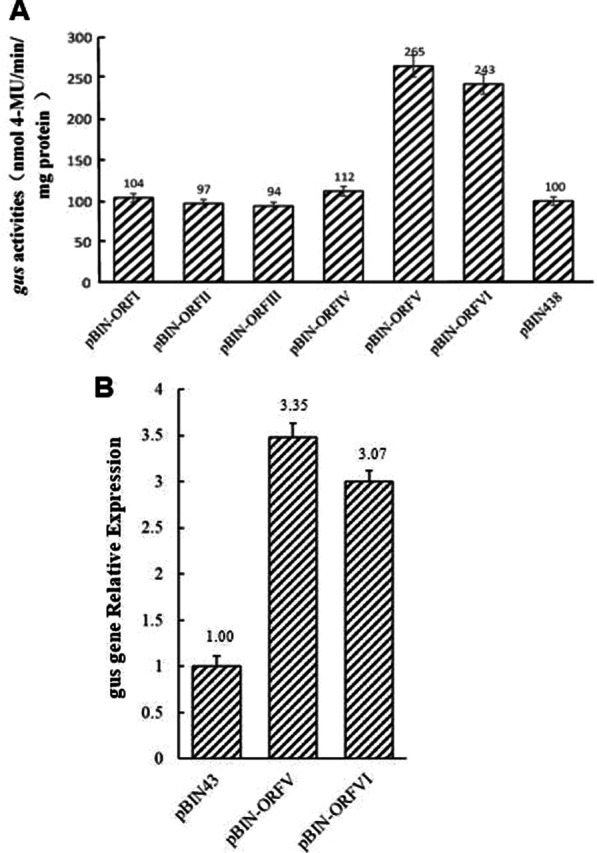
Effects of SVBV-encoded proteins on foreign gene expression driven by the SVBV SP1 promoter. **A** Analysis of GUS activity in *N. benthamiana* leaves..*Agrobacterium* cells harboring the pINT-SP1 vector were co-inoculated into *N. benthamiana* leaves with *Agrobacterium* cells harboring the pBIN-ORFI, pBIN-ORFII, pBIN-ORFIII, pBIN-ORFIV, pBIN-ORFV, pBIN-ORFVI, or pBIN438 (control) vector. The inoculated leaves were harvested at 64 hpi and then analyzed for GUS activity using fluorometric assays. Standard errors were determined using the LSD method. The biochemical expression assay in leaves was repeated thrice and average readings are represented. Statistical analysis showed a *P* value of < 0.05, indicating high significance. **B** Relative expression levels of the *GUS* gene in different tobacco plants. Total RNA was extracted from leaves harvested from tobacco plants separately co-inoculated with the pINT121 and pBIN-ORFV, pBIN-ORFVI, or pBIN438 (negative control) vector. Relative *GUS* mRNA levels were determined by RT-qPCR using *GUS* gene-specific primers. The relative expression level of the tobacco β-actin gene was used as an internal control. Standard errors were determined using the LSD method. The biochemical expression assay in leaves was repeated thrice and average readings are represented. Statistical analysis showed a *P* value of < 0.05, indicating high significance

SVBV gene-specific primers (Table 1) were used to amplify full-length ORFs I, II, III, IV, V, and VI from the Chinese SVBV isolate, yielding PCR products with lengths of 986, 485, 320, 1415, 2099, and 1556 bp, respectively. The resulting PCR fragments were individually cloned into the expression vector pBIN438, harboring the 35S promoter, to generate pBIN-ORFI, pBIN-ORFII, pBIN-ORFIII, pBIN-ORFIV, pBIN-ORFV, and pBIN-ORFVI, respectively. These expression vectors were then individually transformed into *Agrobacterium* and co-inoculated with *Agrobacterium* harboring pINT-SP1 into *N. benthamiana* leaves. Leaves co-inoculated with *Agrobacterium* harboring pINT-SP1 and pBIN438 were used as controls. The results showed that the GUS activities in leaves co-inoculated with *Agrobacterium* harboring pINT-SP1 and pBIN-ORFV or *Agrobacterium* harboring pINT-SP1 and pBIN-ORFVI were 2.7- and 2.4-fold greater than those in leaves co-inoculated with *Agrobacterium* harboring pINT-SP1 and pBIN438 (Fig. 5A). Furthermore, GUS activities in leaves co-inoculated with *Agrobacterium* harboring pINT-SP1 and pBIN-ORFI, pINT-SP1 and pBIN-ORFII, pINT-SP1, pBIN-ORFIII, pINT-SP1, and pBIN-ORFIV were similar to those in the leaves co-inoculated with *Agrobacterium* harboring pINT-SP1 and pBIN438. These results indicated that SVBV ORF V and VI could enhance foreign gene expression driven by the SVBV SP1 promoter, whereas other SVBV ORFs had no significant effect on foreign gene expression driven by this promoter. We further assessed GUS mRNA levels in different transgenic plants using RT-qPCR of total RNA isolated from *N. benthamiana* leaves co-inoculated with the pBIN-ORFV, pBIN-ORFVI, and pINT-121. Leaves co-inoculated with *Agrobacterium* harboring pBIN438 and pINT-121 were used as controls. The results showed that GUS mRNA accumulated to a significantly higher level in pBIN-ORF/pINT-121 and pBIN-ORFVI/pINT-121 than those in leaves co-inoculated with *Agrobacterium* harboringin pBIN438/pINT-121 (Fig. 5B).

## Discussion

### Transient gene expression driven by the SVBV SP1 promoter

In this study, we compared the strength of gene expression driven by the full-length SP1 promoter with several deletion mutants. Our results indicated that the SP2 promoter, which contained 324 bp, one GA motif, and two CAAT boxes, drove the expression of *GFP* and *GUS* genes in plants. This finding suggested that this 324-bp fragment was likely the minimal region for promoter activity. The SP3 promoter retained the minimal promoter region and several upstream regulatory elements (e.g., five CAAT boxes and two TAAT boxes) and yielded expression levels that were approximately threefold greater than those generated from the SP2 promoter. This result indicated that the five CAAT boxes and two TAAT boxes in the SP3 promoter had a significant influence on promoter activity. The SP1 promoter contained two additional CAAT boxes compared with the SP3 promoter at its 5′ end and its effects on gene expression were about threefold stronger than those of the SP3 promoter, indicating that the additional two CAAT boxes at the 5′ end of the SP1 promoter also had a strong effect on the SVBV promoter. The TATA box sequence 40 bp upstream of the transcription initiation site (+ 1) is a known binding site for RNA polymerase II [24]. Importantly, we then deleted the 30 nucleotides at the 3′ end of the SP1 promoter and found that this deletion mutant (i.e., SP4) had much lower promoter activity than the SP1 promoter. This result suggested that deletion of this 3′ sequence may have affected the binding of RNA polymerase II, thereby leading to weak promoter activity.

### SVBV promoter-driven stable gene expression in plants

The CAAT box and TAAT box are core promoters, also known as basal promoters [25–28]. The SP1 promoter cloned in this study possessed seven CAAT boxes and four TAAT boxes, and its activity was likely controlled by these regulatory elements. Notably, constitutive expression driven by promoters may be controlled by specific regulatory elements. For example, the regulatory element as-2 box in the CaMV 35S promoter could be induced by light to promote the expression of genes in leaf photosynthetic tissues and roots [29, 30]. The GATA box in the cassava vein mosaic virus promoter has been shown to regulate gene expression in green tissues [31, 32]. The as-2 box and GATA box sequences were also present in the SVBV SP1 promoter. We speculate that constitutive gene expression driven by the SP1 promoter may also be modulated by these two regulatory elements. In this study, our RT-qPCR results showed that the expression levels of the *GUS* reporter gene driven by the SP1 promoter were significantly higher than those driven by the CaMV 35S promoter.

### Effects of other SVBV-encoded proteins on SVBV SP1 promoter activity

Both SVBV and CaMV are members of the genus *Caulimovirus*. Based on the classification of CaMV ORFs, ORF I of SVBV encodes a protein necessary for virus intercellular movement, ORF II encodes a protein involved in aphid vector transmission, ORF III encodes a non-sequence-specific DNA-binding protein, ORF IV encodes a capsid protein that is important for virion formation, ORF V encodes a reverse transcriptase, and ORF VI encodes a viral determinant of disease symptoms and host range. The function of the protein encoded by ORF VII remains unclear [33–35]. Our results showed that co-inoculation of the ORF V or ORF VI vector with the SP1 promoter-driven expression vector resulted in enhanced *GUS* gene expression, whereas the other ORF expression vectors had no such effect.

CaMV first transcribes RNA with the virion DNA as the template and then reverse transcribes double-stranded DNA using the above RNA as the template [36]. ORF V is a reverse transcriptase gene, the protein encoded by ORF V plays a role in the process of virus reverse transcription to synthesize DNA from RNA. ORF V promote the transcriptional activity of the promoter, perhaps the reverse transcriptase protein encoded by ORF V combines with some cis-elements of SVBV promoter to promote transcription, or interacts with some transcription factors to promote transcription, thus increasing the expression of Gus reporter gene.

The CaMV gene ORF VI encodes a multifunctional protein that has a transactivation function. As a transactivator (TAV), the ORF VI protein forms dense cell inclusions in the virus-infected cytoplasm [37, 38]. The translation of ORF VII followed by ORF VI is reinitiated by CaMV 35S RNA through the ribosome diverting mechanism [39], with the assistance of TAV, increasing translation by two–threefold. The TAV encoded by SVBV ORF VI [40] may enhance the transcriptional activity of the promoter and stimulate the expression of the reporter gene *GUS*.

## Conclusions

The CaMV 35S promoter is currently the most widely used expression promoter during stable plant transformation. However, the SP1 promoter from the Chinese SVBV isolate described in this study was shown to be a stronger promoter than the CaMV 35S and FLt-US promoters and may be more useful for the production of stable transgenic plants.

## Supplementary Information

**Additional file 1**. Table S1. The comparison of the functional elements between SVBV promoter and CaMV 35S promoter. The comparison is analyzed by PlantCARE (http://bioinformatics.psb.ugent.be/webtools/plantcare/html/) (A) and (B).

## Data Availability

All data generated or analyzed during this study are included in this published article.

## Abbreviations

CaMV: Cauliflower mosaic virus
CP: Capsid protein
FLt-US: SVBV US strain promoter
GFP: Green florescence protein
GUS: *β*-Glucuronidase
kb: Kilobases
MUG: Methylumbelliferyl-*β*-D-glucuronide
ORFs: Open reading frames
qRT-PCR: Quantitative RT-PCR
SVBV: Strawberry vein banding virus
TAV: Trans-activator
