# Supplementary material for: Functional analysis of a viral promoter from a strawberry vein banding virus isolate from China
Source: Virol J. 2022 Mar 31;19:60. doi: 10.1186/s12985-022-01778-2 (PMC8974135; doi:10.1186/s12985-022-01778-2)
Supplement: Supplementary file 1 — Additional file 1. Table S1. The comparison of the functional elements between SVBV promoter and CaMV 35S promoter. The comparison is analyzed by PlantCARE (http://bioinformatics.psb.ugent.be/webtools/plantcare/html/) (A) and (B). [file 12985_2022_1778_MOESM1_ESM.docx]

| **The comparison table**  SVBV (HE681085) promoter 1017 bp. Transcription initiation site (+1) in 7264 bp | | | | |
| --- | --- | --- | --- | --- |
|  | Sequence | Number | Position | Function |
| TATA-box | TTTTA or TATATAA | 2 | -40，-62 | core promoter element of transcription start |
| CAAT-box | CAAT or CAAAT | 7 | -342，-444，-516，-710，-755，-918，-933 | common cis-acting element in promoter and enhancer regions |
| GA-motif | AAAGATGA | 1 | -257 | part of a light responsive element |
| as-2-box | GAAAATGATG | 1 | -666 | involved in shoot-specific expression and light responsiveness |
| TCA-element | CAGAAAAGA | 1 | -260 | cis-acting element involved in salicylic acid responsiveness |
| TC-rich repeats | GTTTTCTTCC | 1 | -83 | cis-acting element involved in defense and stress responsiveness |
| MBS | TAACTG | 1 | -556 | MYB binding site involved in drought-inducibility |
| CCGTCC-box | CCGTCC | 1 | -545 | cis-acting regulatory element related to meristem specific activation |
| GATA-motif | AAGATAAGACT | 1 | -158 | part of a light responsive element |
|  |  |  |  |  |
|  |  |  |  |  |
| CaMV (V00141) 35S promoter 1017 bp. Transcription initiation site (+1) in 7434 bp | | | | |
|  | Sequence | Number | Position | Function |
| TATA-box | TATA or ATATAA | 2 | -646，-30 | core promoter element of transcription start |
| CAAT-box | CAAT or CAAAT | 9 | -917，-817，-739，-721，-510，-442，-347，-238，-63 | common cis-acting element in promoter and enhancer regions |
| G-box | CACGTC or CACGAC | 2 | -405， -121 | cis-acting regulatory element involved in light responsiveness |
| as-1-motif | TGACG | 3 | -862，-,82，-70 | cis-acting regulatory element involved in the MeJA-responsiveness |
| I-box | GGATAAGGTG | 1 | -870 | part of a light responsive element |
| GT1-motif | GGTTAA | 1 | -703 | light responsive element |
| ARE | AAACCA | 1 | -588 | cis-acting regulatory element essential for the anaerobic induction |
| TCT-motif | TCTTAC | 1 | -452 | part of a light responsive element |
| CGTCA-motif | CGTCA | 1 | -421 | cis-acting regulatory element involved in the MeJA-responsiveness |
| TGA-box | TGACGTAA | 1 | -82 | part of an auxin-responsive element |
|  |  |  |  |  |
| Analysed by PlantCARE (http: //bioinformatics.psb.ugent .be/webtools/plantcare/html/) | | | | |
